# Transseptal access complication: Fractured and trapped wire segment

**DOI:** 10.1016/j.hrcr.2026.06.042

**Published:** 2026-07-22

**Authors:** Matthew Brennan, Kartik Gupta, Brian La Starza, Lama Al Jebbawi, Pedro Villablanca, Joshua Greenberg

**Affiliations:** 1 Department of Medicine, Henry Ford Hospital, Detroit, Michigan; 2 Department of Cardiology, Henry Ford Hospital, Detroit, Michigan; 3 Division of Cardiac Electrophysiology, Department of Internal Medicine, Henry Ford Health, Detroit, Michigan; 4 Structural Heart Disease, Division of Cardiology, Henry Ford Health, Detroit, Michigan

**Keywords:** Transseptal puncture, Pulmonary vein isolation, Pigtail guidewire, Wire fracture, Wire fragment, Atrial septal defect device

**Key Teaching Points**

• The unique design of pigtail guidewires may make them susceptible to fracture when advanced against resistance at the interatrial septum. This fracture may lead to septal entrapment.
• When inserting the transseptal guidewire, advance the dilator to the mid-left atrium (LA) under intracardiac echocardiography guidance to ensure LA access.
• Manufacturer testing revealed that guidewire fracture risk is highest when the wire is exposed and the sheath-dilator is advanced against the interatrial septum.
• When retrieval of a wire fragment is not feasible, the use of an atrial septal defect closure device may help mitigate the risk of embolization.

## Introduction

Transseptal access for left-sided procedures is a routine procedure with rare complications. We describe a rare complication of a fractured and entrapped pigtail wire tip in the interatrial septum. We also describe a novel strategy of using an atrial septal closure device to reduce risk of embolization.

## Case report

### Background

A 65-year-old man with persistent (stage 3b) atrial fibrillation and atrial flutter presented for cavotricuspid isthmus ablation and pulmonary vein isolation (PVI) with radiofrequency (RF). He was intubated under general anesthesia, and ultrasound-guided access was obtained in the right femoral vein. Using the CARTO^TM^ 3 electroanatomic mapping system (Biosense Webster, Irvine, CA), a right atrial matrix was created with a mapping catheter, and all 4 cardiac chambers were identified via intracardiac echocardiography (ICE) integration. A decapolar catheter was inserted into the coronary sinus and cavotricuspid isthmus ablation was completed, with confirmed bi-directional block.

### Transseptal access

An 8.5-Fr VIZIGO guiding sheath with dilator and transseptal RF needle (Baylis NRG^TM^) were advanced to the superior vena cava under ICE and fluoroscopic guidance. The sheath was dragged from the superior vena cava to the septum when a downward and leftward jump was seen on fluoroscopy. Engagement of the fossa ovalis was confirmed by tenting seen on ICE. The RF transseptal needle was advanced to the distal tip of the sheath and crossed the septum after a short delivery of RF energy. The sheath was advanced over the needle into the left atrium, and access was confirmed by visualizing agitated saline bubbles in ICE. The needle was exchanged for a spring-coil pigtail TORAYGUIDE™ guidewire (TRG-25175, Toray Medical Co., Ltd., Tokyo, Japan). During the exchange, poor blood return was noted from the sheath, suggesting a possible loss of free communication with the left atrium. The pigtail guidewire was advanced and exposed, but resistance was met at the septum level, preventing further advancement. Upon removal of the guidewire, it was clear that the spring-coil tip had unraveled (Figure 1). ICE and fluoroscopy revealed the distal guidewire tip had fractured and was retained within the septum (Figure 2). Several different angiographic views showed a stable, fixed wire fracture within the septum, without migration (not shown). ICE visualized the mobile end of the fragment on the left atrial side of the IAS. Although the patient was under sedation, we had a multidisciplinary discussion with board-certified cardiologists trained in advanced cardiac imaging and structural heart interventions. We decided that the wire fracture was sufficiently stable to allow completion of the procedure and to place an atrial septal defect (ASD) occluder at the end of the case. A second transseptal access was obtained at a position inferior to the first. Access was obtained without complication using a new ProTrack^TM^ Pigtail Wire to maintain left atrial access.

**Figure 1:**
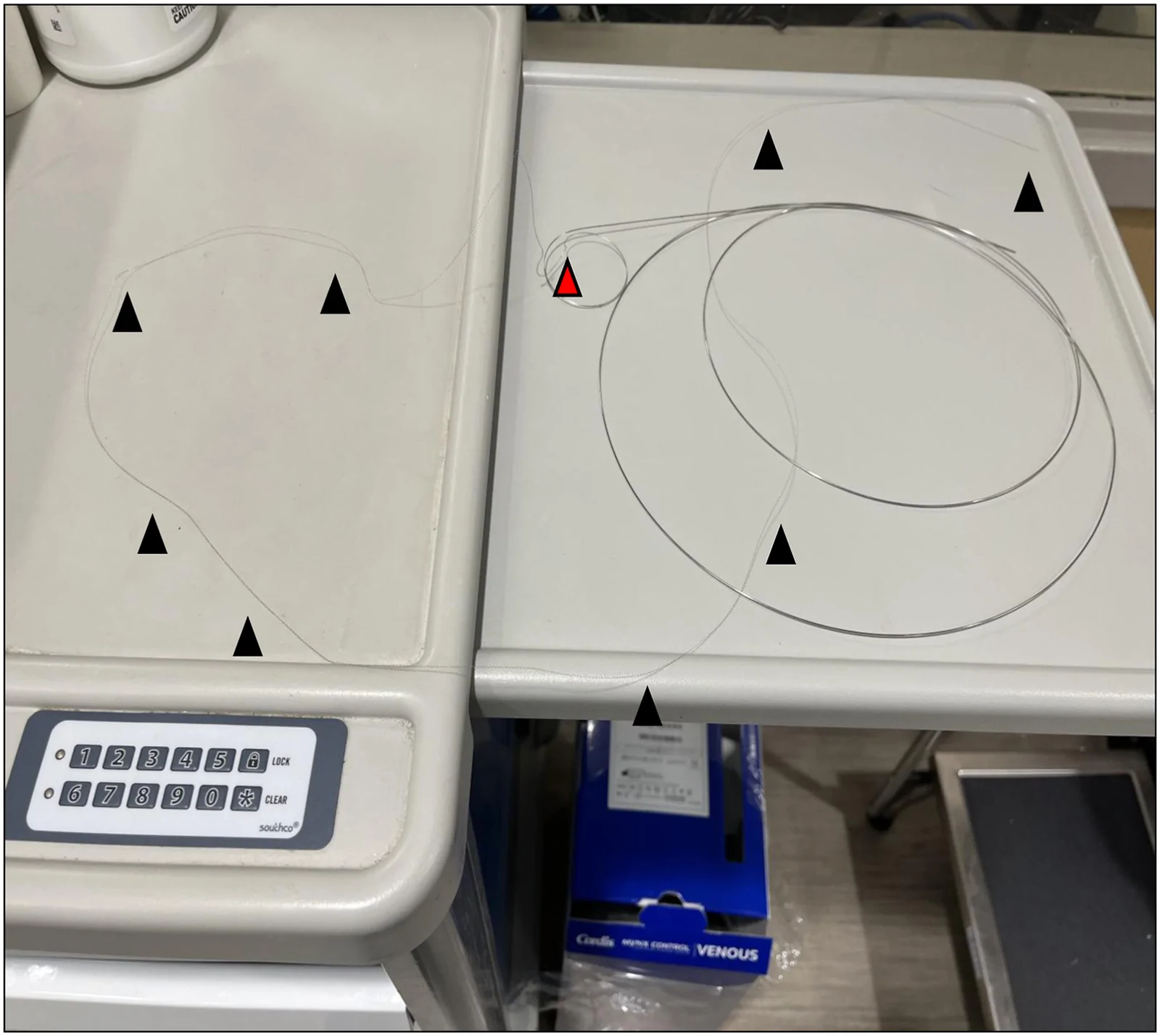
Uncoiled pigtail wire segment after removal from sheath (*red arrow* marks the transition and *black arrows* mark the stripped segment).

**Figure 2:**
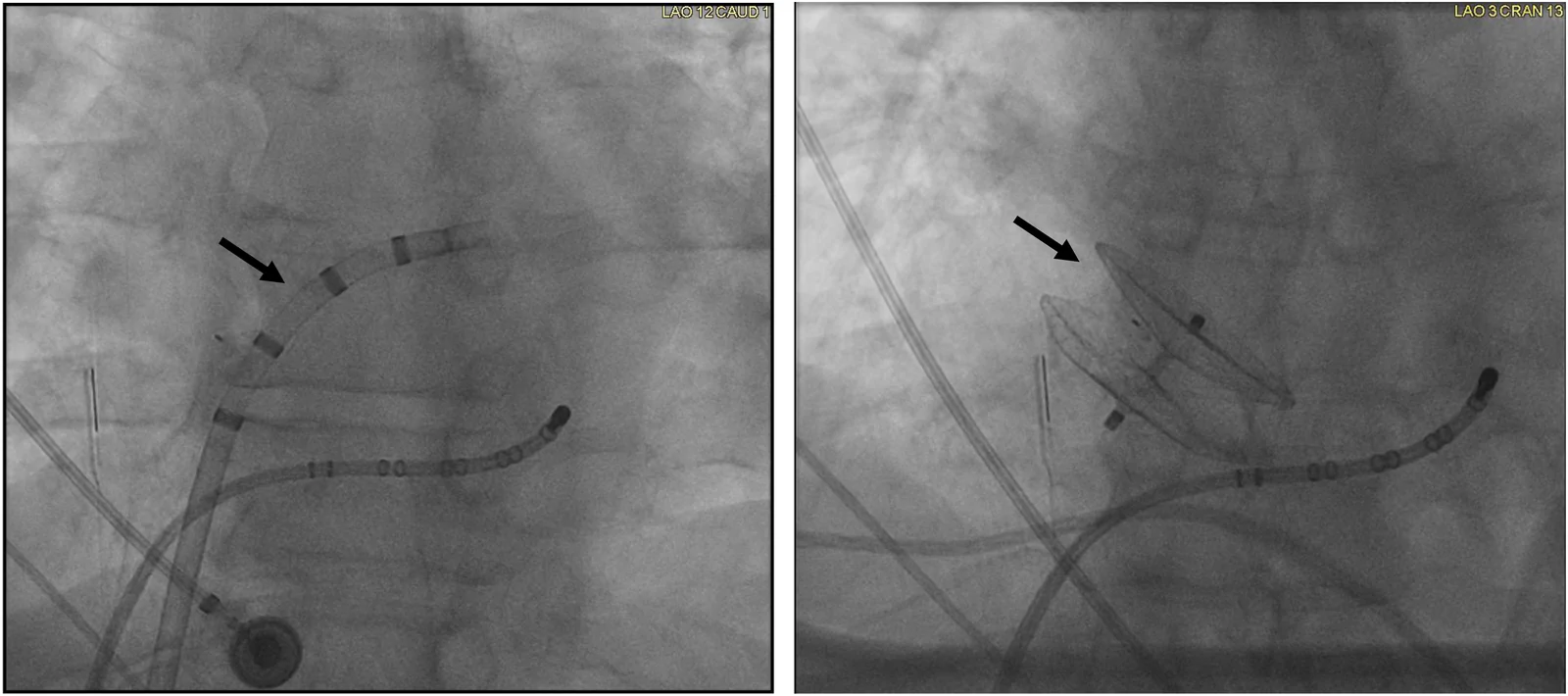
Left anterior oblique radiographic view showing trapped solder tip (*black arrow*) of the guidewire on the left side of the septum (*left*) and anteroposterior cranial view after deployment of atrial septal defect occluder (*right*). There is an intracardiac echocardiography catheter in the right atrium and a coronary sinus catheter.

### PVI and ASD closure device

The PVI was completed as planned. The wire fragment remained lodged in the septum through the procedure. To prevent embolization of the trapped fragment or *in situ* thrombus formation, a 35 mm Amplatzer™ ASD device was implanted to cover the wire fragment. ICE and fluoroscopy were used to confirm the position of the wire fragment, trapped within the ASD device (Figure 2). Reversal of intraoperative heparin was not given, and the patient recovered without complication. At 6 months, the patient had experienced no thromboembolic events and a Holter monitor demonstrated normal sinus rhythm without recurrence of atrial fibrillation.

## Discussion

This is the first report of fracture of the TORAYGUIDE ™ guidewire, and its distinctive design characteristics warrant closer examination. The TRG-25175 TORAYGUIDE ™ is a 175 cm stainless steel 0.025-inch core wire with a 140 mm spring coil (Figure 3).

**Figure 3:**
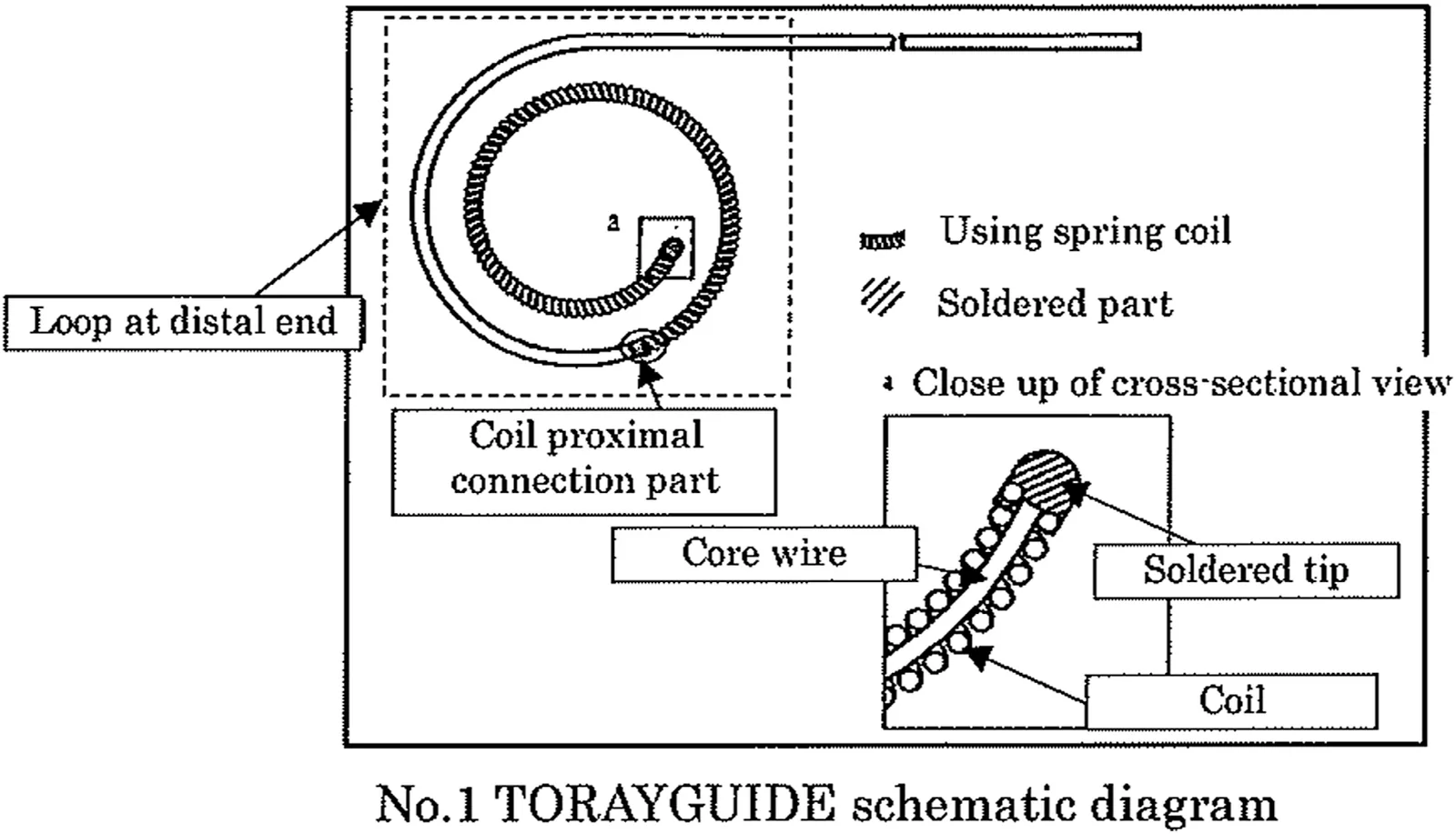
Schematic diagram of structure of TORAYGUIDE wire. TRG-25175 is made of a solid 304 stainless steel body with a length of 175 cm, a diameter of 0.025 inch, and a distal spring coil of 140 mm.

An investigation of the returned wire by Toray Medical Co. Ltd. had the following findings: (1) the soldered tip was missing; (2) core wire: there was a loop-shaped kink in the core wire 76 mm from the coil’s proximal connection part towards the distal end (Figure 4A and 4B), and the core wire was fractured 134 mm from the coil’s proximal connection part towards the distal end (Figure 4A and 4B); and (3) coil: the coil was loosened from the core wire and extended ∼1.3 m from its proximal connection part towards the distal end, with a fracture at its midpoint (Figure 4A and 4B).

**Figure 4:**
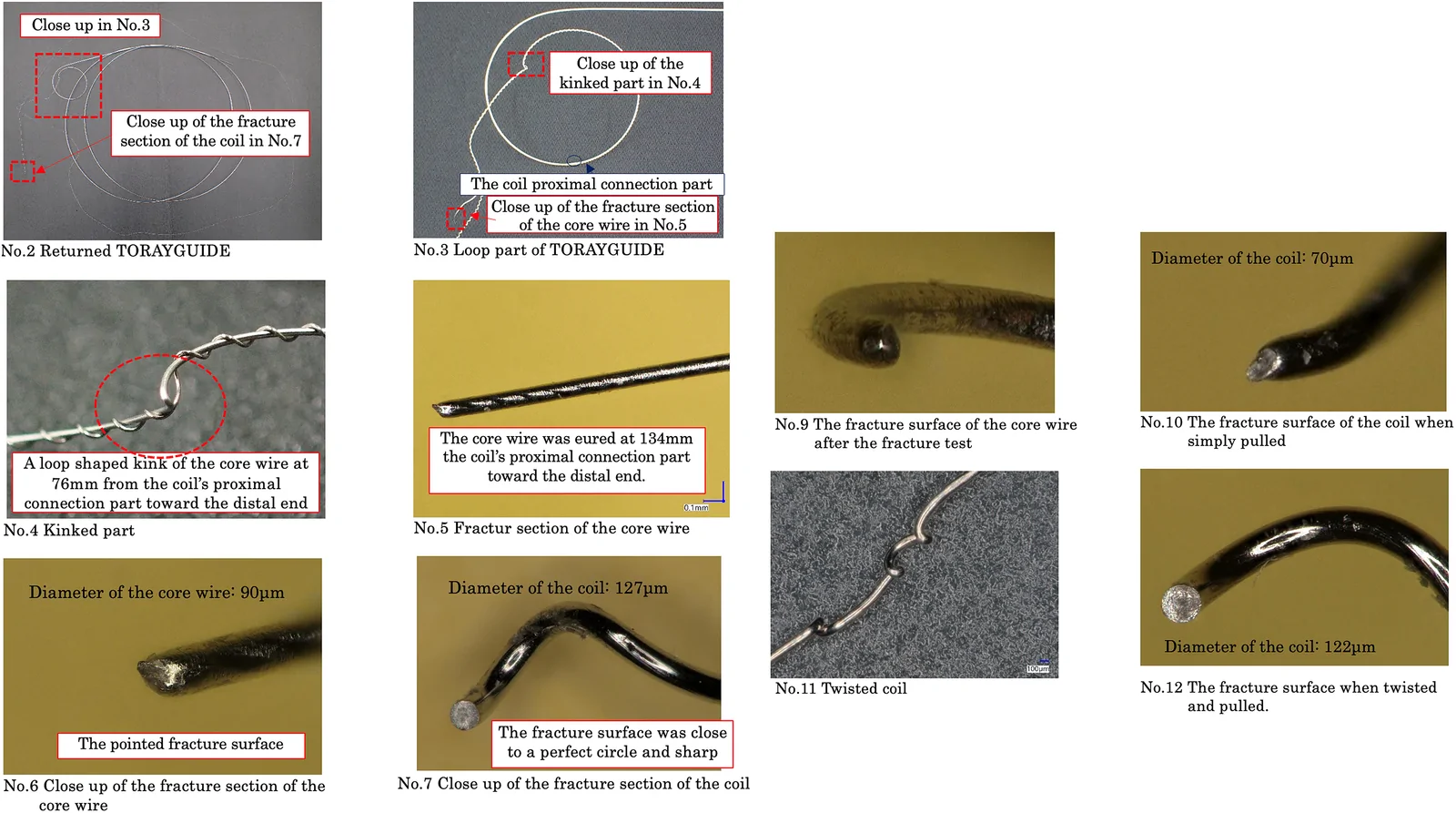
Investigation from Toray Medical Co., Ltd (reproduced with permission).

Toray Medical Co. Ltd. performed in-house testing of the TORAYGUIDE^TM^ pigtail guidewire using a chicken breast to represent the septum (Figure 5). During the test, the wire was exposed 3 mm distal to the septal crossing dilator (Swartz^TM^, SL1^TM^, Abbott Laboratories, Chicago, IL). With the dilator pressed against the chicken breast, the wire was pulled towards the proximal side. It was observed that the core wire fractured with <1 kilogram-force, which is the lower limit specification for the tensile strength. The soldered tip was trapped between the chicken breast and the dilator. Thus, it is possible that during wire exchange, the sheath got retracted with the tip barely crossing the septum. When the pigtail wire was exposed, only part of the soldered tip crossed the septum into the left atrium, and the remaining wire got trapped. When resistance was encountered, the wire was retracted, and the coil unraveled from the core wire. The coil was also tested. When it was twisted, it fractured under less than 0.2 kilogram-force. Our experience reiterates the importance of ensuring that the distal end of the sheath is completely in the left atrium during all wire exchanges. This can be done both with ICE and by checking for brisk blood return during exchange.

**Figure 5:**
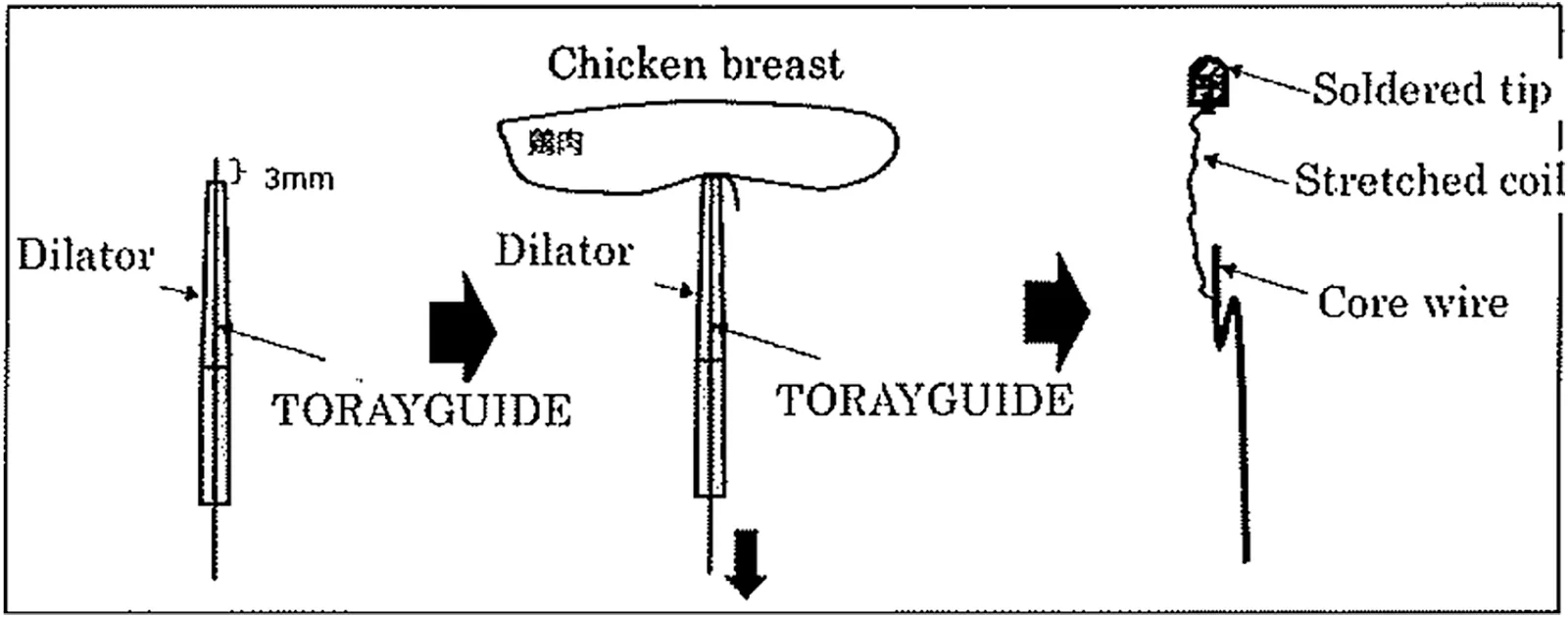
Verification testing from Toray Medical Co., Ltd (reproduced with permission) suggested that if the guidewire is inserted with its tip just distal to the dilator and advanced against the interatrial septum, there could be a fracture of the core wire under a force of <1 kilogram force (lower limit of specified tensile strength).

The operators in this case had performed >200 transseptal accesses. To the best of our knowledge, there is only 1 other reported case of guidewire fracture during transseptal puncture, which involved the fracture and unraveling of ProTrack^TM^ Pigtail Wire.^1^ In this other case, the coil had unraveled from the corewire and got trapped within a thickened interatrial septum. The unraveled coil had completely detached from the core wire and was only visible in ICE and not fluoroscopy. Although the wire was not sent for detailed analysis, the authors similarly hypothesized that the likely mechanism was loss of left atrial access and advancement of the pigtail wire against the right side of the fossa ovalis.

The instructions for use manual for the TORAYGUIDE^TM^ pigtail guidewire specifically warns to “never push, auger, or withdraw a guidewire that meets resistance.”^2^ In our case, the forensic analysis of the wire and radio-opaque density seen on fluoroscopy confirms that the retained fragment was the soldered tip. Although the soldered tip was secured within the septum with minimal motion in fluoroscopy, we chose to deploy an ASD closure device to minimize risk of embolization. Future transseptal access can still be obtained through the patent foramen ovale device. The deployment was secured under ICE visualization. These images were unfortunately not saved.

## Conclusion

Pigtail guidewires may be vulnerable to fracture when advanced against resistance at the septum. Manufacturer testing revealed that the risk of fracture is highest when the guidewire is slightly exposed and the sheath is advanced against the septum. This case highlights practical strategies to prevent recurrence, including verification of left atrial access and avoidance of sheath manipulation when guidewire resistance is encountered. In cases where fragment retrieval is not feasible, deployment of an ASD device is a viable strategy to mitigate the risk of embolism.

## Disclosures

The authors have no conflicts of interest to disclose.

## References

[1] Lee J.C., Gallagher R. (2019). Extraction of radiolucent fractured wire components using intracardiac ultrasound during pulmonary vein isolation procedure. HeartRhythm Case Rep.

[2] Toray Medical Co Ltd. TORAYGUIDE guidewire instructions for use. Tokyo, Japan. https://www.toray.us/assets/pdf/IFU_TRG.pdf.

